# Performance of the G4 Xpert^®^ MTB/RIF assay for the detection of *Mycobacterium tuberculosis* and rifampin resistance: a retrospective case-control study of analytical and clinical samples from high- and low-tuberculosis prevalence settings

**DOI:** 10.1186/s12879-016-2039-4

**Published:** 2016-12-20

**Authors:** Nila J. Dharan, Robert Blakemore, Alex Sloutsky, Devinder Kaur, Richard C. Alexander, Minoo Ghajar, Kimberlee A. Musser, Vincent E. Escuyer, Marie-Claire Rowlinson, Susanne Crowe, Rafael Laniado-Laborin, Eloise Valli, Pamela Nabeta, Pamela Johnson, David Alland

**Affiliations:** 1 Department of Medicine, New Jersey Medical School, Rutgers, The State University of New Jersey, 185 South Orange Avenue, MSB I-689, Newark, NJ 07103 USA; 2 University of Massachusetts Medical School, Worcester, MA USA; 3 Orange County Health Care Agency, Santa Ana, CA USA; 4 Wadsworth Center, New York State Department of Health, Albany, NY USA; 5 Florida Department of Health, Jacksonville, FL USA; 6 Clínica y Laboratorio de Tuberculosis, Hospital General Tijuana, Tijuana, Mexico; 7 Foundation for Innovative New Diagnostics, Geneva, Switzerland; 8 Cepheid, Sunnyvale, CA USA; 9 Current address: Sackler School of Graduate Biomedical Science – Tufts University, Boston, MA USA

**Keywords:** Tuberculosis, Diagnostics, GeneXpert, Xpert, G4

## Abstract

**Background:**

The Xpert^®^ MTB/RIF (Xpert) assay is a rapid PCR-based assay for the detection of *Mycobacterium tuberculosis* complex DNA (MTBc) and mutations associated with rifampin resistance (RIF). An updated version introduced in 2011, the G4 Xpert, included modifications to probe B and updated analytic software.

**Methods:**

An analytical study was performed to assess Xpert detection of mutations associated with rifampin resistance in rifampin-susceptible and -resistant isolates. A clinical study was performed in which specimens from US and non-US persons suspected of tuberculosis (TB) were tested to determine Xpert performance characteristics. All specimens underwent smear microscopy, mycobacterial culture, conventional drug-susceptibility testing and Xpert testing; DNA from isolates with discordant rifampin resistance results was sequenced.

**Results:**

Among 191 laboratory-prepared isolates in the analytical study, Xpert sensitivity for detection of rifampin resistance associated mutations was 97.7% and specificity was 90.8%, which increased to 99.0% after DNA sequencing analysis of the discordant samples. Of the 1,096 subjects in the four clinical studies, 49% were from the US. Overall, Xpert detected MTBc in 439 of 468 culture-positive specimens for a sensitivity of 93.8% (95% confidence interval [CI]: 91.2%–95.7%) and did not detect MTBc in 620 of 628 culture-negative specimens for a specificity of 98.7% (95% CI: 97.5%–99.4%). Sensitivity was 99.7% among smear-positive cases, and 76.1% among smear-negative cases. Non-determinate MTBc detection and false-positive RIF resistance results were low (1.2 and 0.9%, respectively).

**Conclusions:**

The updated Xpert assay retained the high sensitivity and specificity of the previous assay versions and demonstrated low rates of non-determinate and RIF resistance false positive results.

**Electronic supplementary material:**

The online version of this article (doi:10.1186/s12879-016-2039-4) contains supplementary material, which is available to authorized users.

## Background

The Xpert^®^ MTB/RIF (Xpert) assay [1, 2] detects the presence of *Mycobacterium tuberculosis* complex DNA (MTBc) and mutations associated with resistance to rifampin (RIF) in clinical samples in under two hours. Xpert was endorsed by the World Health Organization (WHO) in December 2010 [2, 3] and recent meta-analyses describe the assay’s excellent performance characteristics in the field [4, 5]. Since the release of Xpert for use, several modifications have been made to improve assay performance. The updated version, the G4 Xpert, includes modifications to one of the five *rpoB*-specific assay probes (probe B) and to the analytic software settings with the goal of decreasing both false positive RIF resistance results and the >5% non-determinate result rate reported in some settings [6].

Although the updated G4 Xpert assay was available for use in December 2011 [6], most published Xpert performance reports were conducted using previous versions of the assay [4]. In addition, few studies have reported the performance of the G4 Xpert on samples from both high- and low-tuberculosis (TB) prevalence populations. Here we present data on G4 Xpert assay performance from analytical testing and multi-site clinical studies in various settings, including settings with a low prevalence of TB.

## Methods

### Study design and specimen inclusion criteria

#### Analytical testing of rifampin resistance detection performance

Xpert performance of RIF resistance detection was evaluated using 200 unique clinical isolates spiked into pooled human sputum. The isolates were selected from the specimen bank at the Massachusetts Supranational Tuberculosis Reference Laboratory at the University of Massachusetts Medical School and had been obtained from standard of care (SOC) sputum specimens from individuals in Russia, Peru, Hong Kong, Haiti, and USA. Isolates were selected based on phenotypic drug susceptibility test results. One hundred RIF susceptible isolates, 100 RIF resistant isolates and an additional 50 aliquots of MTB culture-negative pooled human sputum were randomized and blinded for testing.

#### Clinical studies

Four clinical studies were conducted to assess Xpert detection of MTBc and RIF resistance in specimens from patients 18 years of age or older with suspected TB. Clinical study 1 (CS1) included four collections of archived MTB culture-positive and -negative specimens collected from non-US regions as part of research studies conducted by the Foundation for Innovative New Diagnostics [7]. Subject inclusion criteria were: 1) pulmonary TB symptoms (see Additional file 1); and 2) no anti-TB medication in the 60 days prior to sample collection. Xpert testing was performed at New Jersey Medical School (NJMS).

Clinical study 2 (CS2) specimens consisted of archived MTB culture-positive and culture-negative sputum specimens that were leftover from SOC evaluations of US patients with suspected TB illness. Xpert testing was done at the State of New York Department of Health in Albany, NY.

Clinical study 3 (CS3) and 4 (CS4) tested up to three prospectively collected leftover SOC specimens from US (CS3) and Mexican (CS4) patients suspected to have TB; the first specimen with sufficient volume was selected for Xpert testing. CS3 Xpert testing was done at the New York State Department of Health in Albany, NY, the Florida Department of Health, Bureau of Public Health Laboratories in Jacksonville, FL, and the Orange County Health Care Agency in Santa Ana, CA. CS4 Xpert testing was performed at the Orange County Health Care Agency in Santa Ana, CA.

See Additional file 1 for further details on study design and specimen inclusion criteria.

### Laboratory testing

In the analytical study, culture-positive isolates were confirmed to be MTBc positive by AccuProbe MTBC Identification Test (Hologic Incorporated, Marlborough, MA). Rifampin susceptibility was determined via the Middlebrook agar proportion method [8] according to each laboratory’s standard operating procedure. Bi-directional sequencing of the *rpoB* core region was performed for all specimens with discordant RIF resistance results. The culture isolates were spiked at low, moderate, or high concentrations into sputa (see Additional file 1) and tested with the Xpert assay. Fifty aliquots of pooled MTB negative human sputum were interspersed randomly during testing as a negative control.

The methods used at each of the clinical study sites for AFB smear, MTB culture, and DST are summarized in Table 1. Samples in all studies were tested by Xpert according to the package insert instructions [9]. All frozen specimens were stored at -70 °C and all prospectively collected samples were stored per the sample storage constraints described in the package insert. If multiple samples from each patient were available, the first specimen with sufficient volume for testing was used. Duplicate specimen enrollment for the same patient for Xpert testing was not allowed. Bi-directional sequencing of the *rpoB* core region was performed on MTB culture-positive isolates with discordant Xpert MTB or RIF susceptibility results. No sequencing was performed for MTB culture-negative specimens with discordant Xpert results. Sequencing of concordant samples was omitted. Sequencing was performed in Borstel Germany for CS1, at the NY State Department of Health for CS2 and CS3, and at the University of Massachusetts Medical School for CS4.

**Table 1:** AFB smear, culture, TB identification and DST methods for each clinical study

Study number	Geographical location/collection site	Xpert MTB/RIF Assay Testing Site	Specimen Used for Xpert MTB/RIF Assay Testing^a^		Specimen Processing for Smear & Culture	AFB Smear Method on Processed Sputum^a^	Culture Method^a^	TB Identification Method^a^	DST Method^a^
CS1	Peru	NJMS	Archived (Frozen)	Raw Sputum	NALC-NaOH	ZN	LJ and MGIT	CapilliaTB-Neo	LJ Proportions
	South Africa, Vietnam, Bangladesh^b,c^	NJMS	Archived (Frozen)	Raw Sputum	NALC- NaOH	ZN	LJ and MGIT	GenoType^®^ MTBC	MGIT SIRE
CS2	New York	New York	Archived (4 °C)	Pellet	NaOH	ZN	7H10 and MGIT	In house RT-PCR	MB Proportions
CS3	New York	New York	Fresh	Pellet	NaOH	ZN	7H10 and MGIT	In house RT-PCR	MB Proportions^d^
	California	California	Fresh	Pellet	NALC- NaOH	AuRho	7H10 and MGIT	AccuProbe MTBC	MB Proportions^d^
	Florida	Florida	Fresh	Pellet or Raw Sputum	NALC-NaOH			AccuProbe	MB Proportions^d^
						AuO + ZN	LJ and MGIT	AccuProbe MTBC	
CS4	Tijuana, Mexico	California	Fresh	Raw Sputum	NALC-NaOH	AuRho	LJ and MGIT	AccuProbe MTBC^e^	MB Proportions^e^

^a^ *ZN* Ziehl-Neelsen, *AuRho* Auramine Rhodamine, *AuO* Auramine O, *Raw Sputum* direct, not decontaminated/undigested, *Pellet* decontaminated/digested sediment, *MB* Middlebrook, *LJ* Lowenstein-Jensen, *MGIT* BD BBL™ MGIT™ Mycobacteria Growth Indicator Tube, OADC Enrichment, PANTA™ Antibiotic Mixture, (Becton, Dickinson and Company, Sparks, MD), *AccuProbe MTBC* Mycobacterium tuberculosis Complex Culture Identification Test (Hologic, Incorporated, Marlborough MA), *MGIT SIRE* BD BACTEC™ MGIT™ 960 SIRE Kits For the Antimycobacterial Susceptibility Testing of Mycobacterium tuberculosis, (Becton, Dickinson and Company, Sparks, MD), *Capillia TB-Neo* Capilia TB Test Kit (Tauns Laboratories, Numazu, Japan), PNB – p-nitrobenzoic acid, *Genotype® MTBC* GenoType^®^ MTBC (Hain Lifescience GmbH, Nehren, Germany)

^b^Specimens were originally cultured and identified for MTB using the following methods: Capillia TB-Neo, Hain Genotype, Niacin + PNB5 at these sites: Unit for Clinical and Biomedical TB Research, South African Medical Research Council. Durban, South Africa, Pham Ngoc Thach Hospital Laboratory. Ho Chi Minh City, Vietnam, Bangladesh: International Centre for Diarrhoeal Disease Research, Bangladesh. Dhaka, Bangladesh. Raw frozen sputum specimens were available and sent to the National Reference Center (NRC) for Mycobacteria, Supranational Reference Laboratory (SRL) of WHO, Research Center Borstel - Leibniz Center for Medicine and Biosciences, Borstel Germany for culture confirmation and DST and when appropriate bi-directional sequencing

^c^Samples were provided by the Foundation for Innovative Diagnostics (FIND) under their study protocol. FIND staff performed all of the data collection, monitoring and auditing of the study

^d^New York served as the central lab for DST testing and, when appropriate, for bi-directional sequencing

^e^University of Massachusetts Medical School served as the central lab for CS4 TB identification and DST testing

### Clinical study case definitions

AFB smear status was determined using the specimen with a corresponding Xpert result. An MTB positive case was defined as MTB growth on solid or liquid culture from any specimen. An MTB negative case was defined as no MTB growth from any baseline specimen; baseline was defined as collected within seven days of presentation. A case was defined as MTB indeterminate when all cultures were overgrown by non-MTB bacteria or fungi and an MTB positive or negative culture result could not be determined. Phenotypic resistance was determined to be present if 1% or more of the test population grew in the presence of the critical concentration of rifampicin, defined as 1.0 μg/mL.

The GeneXpert software (Version 4.3) reported MTB results as “MTB detected” or “MTB not detected” and RIF resistance results as “MTB detected; RIF resistance detected”, “MTB detected; RIF resistance not detected”, and “MTB detected; RIF resistance indeterminate”. Xpert results of “invalid”, “error” or “no result” were defined as “non-determinate”.

### Statistical analysis

Xpert detection of MTBc DNA was assessed relative to culture; culture indeterminate and Xpert non-determinate specimens were excluded. Xpert detection of mutations associated with RIF resistance was assessed relative to DST; specimens where DST results were not available, MTBc was not detected by Xpert, or MTBc was detected but RIF resistance results were indeterminate were excluded. See Additional file 1 for further details including sample size calculations.

**Fig. 1:**
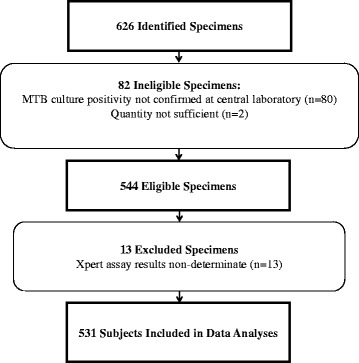
Clinical study 1 specimen accountability

**Fig. 2:**
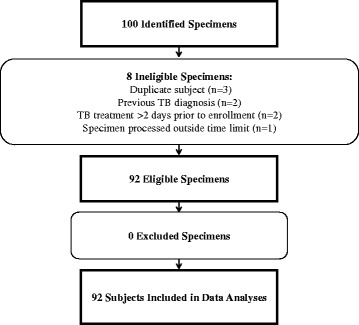
Clinical Study 2 specimen accountability

**Fig. 3:**
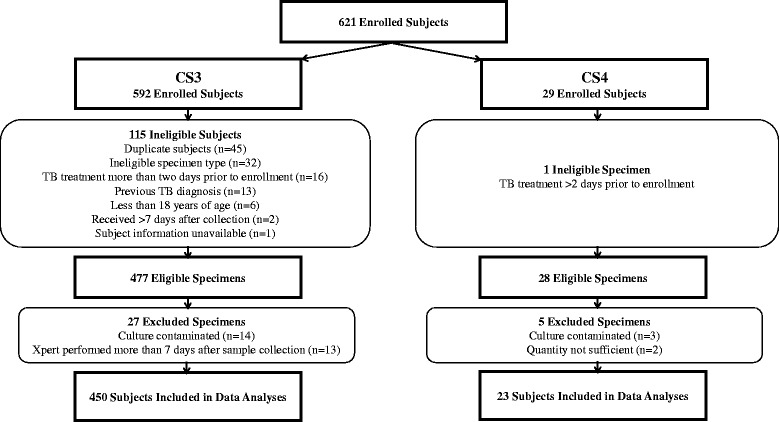
Clinical Studies 3 and 4 specimen accountability

For the clinical studies, any specimens involved in protocol deviations were excluded from analysis (see Figs. 1, 2 and 3). Specimens in CS1 were collected from subjects participating in a research study and specimens tested in CS2-4 were SOC specimens. Data from the four clinical studies were tested for homogeneity across multiple parameters using the Fisher’s Exact Test. A critical p-value was set to 0.01 due to multiple testing in several categories (Bonferroni principle).

## Results

### Specimen inclusion and clinical demographics

#### Analytical study

Of the 200 culture-positive isolates from the specimen bank at the Massachusetts Supranational TB Reference Laboratory identified for inclusion in this study, nine were excluded due to: no culture growth (n = 4); specimen was not inoculated on LJ media (*n* = 4); specimen was lost (*n* = 1); and specimen was from a duplicate subject (*n* = 1). Of the remaining 191, 118 (62%) were from Peru, 64 (34%) were from the U.S., 8 (4%) were from Russia, and one was from an unknown WHO collection site. No other demographic information was collected for this study.

#### Clinical studies

A total of 1,026 specimens were obtained in the four collections of specimens included in CS1. After the 400 specimens in collection two were excluded due to insufficient volume, 626 total specimens remained; of these, 544 were eligible for Xpert testing (Fig. 1). Among these, 358 (66%) were from males and 183 (34%) were from females; the gender of the patient was unknown for three specimens. The average age of the patient at the time of specimen collection was 38 years (range 18-83 years). Two hundred and eighty-eight (53%) patients were from Vietnam, 174 (32%) were from Peru, 79 (15%) were from South Africa, and 3 (0.6%) were from Bangladesh. Thirteen specimens were excluded because Xpert results were non-determinate, resulting in 531 available for inclusion.

Of 100 specimens identified for CS2, 92 were eligible for inclusion in this analysis (Fig. 2). Of these, 51 (55%) were from males, 37 (40%) were from females and the gender of the patient was unknown for 4 (4%). The average age of the patient at the time of specimen collection was 52.6 years (range 18 to 91 years).

CS3 and CS4 were analyzed separately and subsequently combined because specimens in both studies were collected and tested prospectively. Of the 621 subjects initially enrolled (592 in CS3 and 29 in CS4), 473 specimens were eligible for inclusion in the analysis (Fig. 3). Of the enrolled subjects, 274 (60%) were male and 185 (40%) were female; gender was unknown for 14. The age of the subject was known for 118 (24.9%) of study participants; of these, the average age at specimen collection was 52.4 years (range 19 to 88 years). Forty-eight percent (*n* = 227) of the specimens were from California, 25% (*n* = 118) were from New York, 22% (*n* = 105) were from Florida, and 5% (*n* = 23) were from Mexico.

HIV status was either negative or not captured in the clinical studies.

### Xpert performance

#### Analytical study

Of the 191 clinical isolates, 185 were from Xpert positive specimens, four were from MTB not detected specimens, and two were from specimens reported as non-determinate. Xpert reported RIF resistance in 85 of 87 isolates that were RIF resistant by DST for a sensitivity of 97.7% (95% CI: 92.0%-99.4%). The Xpert assay was negative for RIF resistance in 89 of 98 specimens that were RIF susceptible by DST for a specificity of 90.8% (95% CI: 83.5%-95.1%). There were no Xpert RIF indeterminate results. The *rpoB* core region was sequenced for all 15 isolates with discordant Xpert results (Table 2a); Xpert reported RIF resistance in all cases where there was a mutation in the *rpoB* core region associated with RIF resistance and in one case where there was not. Using resistance associated *rpoB* core region mutations to define RIF resistance, Xpert sensitivity for RIF resistance increased to 97.7% (95% CI: 92.0%-99.4%) and specificity to 99.0% (95% CI: 94.4%-99.8%).

**Table 2:** Sequence results for discrepant samples

a. Analytical study			
DST Result	Xpert MTB/RIF assay result	Number	Sequence result^a^
RIF-resistant	MTB not detected	3	MTB
RIF-susceptible	MTB not detected	1	MTB
RIF-resistant	RIF Resistance not detected	2	2 of 2 wild type *rpoB* core
RIF-susceptible	RIF Resistance detected	9	8 of 9 with resistance associated mutations in *rpoB* core: three with 531 ttg, three with 526 tgc, one with 516 gtc, one with 526 ctc 1 of 9 wild type *rpoB* core
b. Combined clinical studies			
Culture/DST result	Xpert MTB/RIF assay result	Number	Bi-directional sequencing
*Smear-positive specimens*			
MTB positive	MTB not detected	1	MTB
MTB negative^b^	MTB detected	1	N/A
*Smear-negative specimens*			
MTB positive	MTB not detected	28	MTB
MTB negative^c^	MTB detected	7	N/A
*Rifampin resistance results*			
MTB positive/RIF- resistant	MTB detected; RIF Resistance NOT detected	1	Resistance associated mutation in *rpoB* core: 531 ttg with evidence of wild type mixture
MTB positive/RIF- susceptible	MTB detected; RIF Resistance detected	4	2 of 4 with resistance associated mutations in *rpoB* core: both with 533 ccg
			2 of 4 with susceptible associated rpoB core: one wild-type and one 514 ttt (silent) mutation.

^a^Culture negativity was based on only one sputum specimen for this subject

^b^Culture negativity classification was based on one sputum specimen for three subjects, two sputum specimens for two subjects and three sputum specimens for two subjects

^c^It is noted that DST did not detect some common mutations associated with rifampin resistance that were identified by sequencing. Several studies have demonstrated that testing the same strains with varying DST methodology can result in different rifampin resistance results [12, 23–25]

#### Clinical studies

The results for all four clinical studies were analyzed together across multiple parameters (see Additional file 1 for results of CS1 alone and poolability analysis).

### Subject demographics

Among 1,096 subjects for which MTB culture results were available, 679 (62%) were male and 396 (36%) were female; gender was unknown for 21 (2%). The subjects were from geographically diverse regions: 542 (49%) were from the US (California, New York and Florida) and 554 (51%) were from outside the US (Vietnam, Peru, South Africa, Mexico and Bangladesh). Of the 542 US specimens, 450 (83%) were collected prospectively and 92 (17%) were from an archived specimen bank; of the 554 non-US specimens, 23 (4%) were prospectively collected and 531 (96%) were from an archived specimen bank. One sputum specimen was collected from 34% of subjects, two from 44%, and 3 from 22%. An Xpert result was obtained with the first specimen collected for 86% of subjects, with the second for 11% of subjects, and with the third for 0.3% of subjects; for 3% of specimens it was unknown which sputum was used for Xpert testing.

### Xpert assay performance for detection of MTB

Overall, Xpert detected MTBc in 439 of 468 total specimens that were culture positive for MTB for a sensitivity of 93.8% (95% CI: 91.2%-95.7%). Xpert did not detect MTBc in 620 of 628 culture-negative specimens for a specificity of 98.7% (95% CI: 97.5%-99.4%). Among smear-positive, culture-positive cases, Xpert detected MTBc in 350 of 351 cases for a sensitivity of 99.7% (95% CI: 98.4%-99.9%). Among smear-negative, culture-positive cases, Xpert detected MTBc in 89 of 117 cases for a sensitivity of 76.1% (95% CI: 67.6%-82.9%). Discordant results are presented in Table 2b.

Among US specimens (low prevalence settings), Xpert sensitivity for detection of MTBc was 91.0% (95% CI: 83.3%-95.4%; *n* = 89) and specificity was 99.3% (95% CI: 98.1%-99.9%; *n* = 453). Among non-US specimens (high prevalence settings), Xpert sensitivity for detection of MTBc was 94.5% (95% CI: 91.7%-96.3%; *n* = 379) and specificity was 97.1% (95% CI: 93.5%-98.8%; *n* = 175). Xpert performance characteristics for detection of MTBc are stratified by sample collection method (535 expectorated, 234 induced, 327 unknown) and type of specimen (606 specimens were raw and 490 were concentrated) in Table 3.

**Table 3:** Xpert MTB/RIF Assay performance vs. MTB culture stratified by specimen collection method and specimen type

	AFB Smear-positive subjects	AFB Smear-negative subjects	All samples
Specimen Collection Method^a^			
Expectorated Sputum (*n* = 535)			
Sensitivity	99.6% (271/272)^b^	79.0% (75/95)^c^	94.3% (346/367)^d^
	95% CI: 97.9 – 99.9%	95% CI: 69.7 – 85.9%	95% CI: 91.4 – 96.2%
Specificity			97.6% (164/168)^b^
			95% CI: 94.0 – 99.1%
Induced Sputum (*n* = 234)			
Sensitivity	100% (15/15)^b^	40.0% (4/10)^c^	76% (19/25)^d^
	95% CI: 79.6 – 100%	95% CI: 16.8 – 68.7%	95% CI: 56.6 – 85.5%
Specificity			99.0% (207/209)^b^
			95% CI: 96.6 – 99.7%
Specimen Type			
Raw Sputum (*n* = 606)			
Sensitivity	99.7% (285/286)^b^	79.4% (77/97)^e^	94.5% (362/383)^b^
	95% CI: 98.0 – 99.9%	95% CI: 70.3 – 86.2%	95% CI: 91.8% - 96.4%
Specificity			97.8% (218/223)^b^
			95% CI: 94.9 – 99.0%
Concentrated Sputum Sediment (*n* = 490)			
Sensitivity	100% (65/65)^b^	60.0% (12/20)^e^	90.6% (77/85)^b^
	95% CI: 94.4 – 100%	95% CI: 38.7 – 78.1%	95% CI: 82.5% - 95.2%
Specificity			99.3% (402/405)^b^
			95% CI: 97.8 – 99.7%

^a^Collection method for 327 specimens was unknown

^b^p-value for difference > 0.1

^c^p-value for difference = 0.014

^d^p-value for difference = 0.004

^e^p-value for difference = 0.084

*AFB* acid fast bacteria

*95% CI* 95% confidence interval

### Xpert assay performance for detection of RIF resistance

MTB positive culture isolates were tested for susceptibility to rifampin using DST and results were compared with Xpert detection of mutations associated with RIF resistance. Of the 1,096 subjects tested by Xpert, 1,082 were included in the analysis. Eight subjects that did not have DST results were excluded. Six of 447 (1.3%, 95% CI: 0.6%-2.9%) specimens that were positive for MTBc and RIF resistance indeterminate by Xpert were also excluded; one of 351 (0.3%, 95% CI: 0.01%-1.6%) smear-positive specimens and five of 96 (5.2%, 95% CI: 2.2%-11.6%) smear-negative specimens.

Of the 1,082 included samples, 627 were culture negative and did not have DST. Of 455 remaining, 21 were RIF resistant and 434 were RIF susceptible by DST. Among the 21 samples with RIF resistance by DST, two were Xpert MTBc negative and one was MTBc detected, RIF resistance not detected. Excluding Xpert MTBc negative samples, Xpert detected mutations associated with RIF resistance in 18 of 19 samples for a sensitivity of 94.7% (95% CI: 75.4%-99.1%). Of 434 samples that were RIF susceptible by DST, 26 were Xpert MTBc negative. Excluding those, Xpert did not report RIF resistance in 404 of 408 samples for a specificity of 99.0% (95% CI: 97.5-99.6%). Four samples were determined to be RIF susceptible by DST and RIF resistant by Xpert. One was tested by Agar Proportions using LJ in Peru and three were tested using MGIT SIRE (BD BACTEC™ MGIT™ 960 SIRE Kits For the Antimycobacterial Susceptibility Testing of Mycobacterium tuberculosis): one in South Africa and two in Vietnam.

Bi-directional sequencing was performed on all isolates from culture-positive specimens with discrepant results (Table 2b). The one Xpert false RIF-susceptible sample was determined to contain a mixture of wild type and mutant *rpoB* core region DNA by sequencing. Three of the four apparent Xpert false RIF resistant samples had *rpoB* core mutations. One of these three was a silent mutation (514ttt) while the other two had *rpoB* core mutations associated with clinically relevant resistance not always identified by phenotypic RIF susceptibility testing [10–13]. Overall, four (0.9%) had false positive rifampin resistance test results; if using sequencing based mitigation, two (0.5%) had false positive rifampin resistance test results.

Of 1,126 tested specimens, 17 were excluded from the sensitivity and specificity analysis due to culture contamination. However, all 17 specimens had valid Xpert results on the first attempt and were included in the calculated non-determinate rate. Of the 1,126 specimens, 24 were non-determinate for the following reasons: 17 probe check failures, two temperature being out of range, two signal losses, one SPC failure, one syringe motion, and one cartridge integrity. Of the 24 non-determinate, 11 were successful on repeat test and 13 were not repeated due to low sample volume. Overall, 13 (1.2%, 95% CI: 0.7% to 2.0%) of 1,126 specimens had a non-determinate Xpert result.

### Positive predictive value (PPV) and negative predictive value (NPV) for Xpert detection of MTB and RIF resistance

The likelihood that a positive test result is a true positive will vary based on the prevalence of TB in the population and whether the AFB smear is positive or negative. A prospective clinical evaluation of Xpert in patients suspected of active TB in the United States resulted in a prevalence of 11.8% and a percentage of AFB-positive smears among MTB culture positive subjects of 75.5%[14]. Hypothetical estimated PPV and NPV of MTB detection using Xpert for different prevalence rates of MTB are shown in Table 4. These calculations are based on the overall sensitivity and specificity observed in all four clinical studies (as above, sensitivity of 99.7% for smear positive specimens, 76.1% for smear-negative specimens and overall specificity of 98.7%).

**Table 4:** Hypothetical Predictive Values of Xpert detection of MTBc vs. MTB Culture

Prevalence of MTB culture positive	Probability of MTB culture positive among		Probability of MTB culture negative among
	Xpert MTB detected AFB Smear Pos.	Xpert MTB detected AFB Smear Neg.	Xpert MTB not detected
1%	82.9	14.4	99.9
2%	90.8	25.4	99.9
3%	93.7	34.1	99.8
4%	95.2	41.0	99.7
5%	96.2	46.8	99.7
10%	98.2	65.0	99.3
*11.8%*	*98.5%*	*69.1%*	*99.2%*
20%	99.2	80.7	98.5
40%	99.7	91.8	96.1
50%	99.8	94.4	94.2

Hypothetical estimated predictive values for the result “MTB Detected, RIF Resistance DETECTED” for different prevalence rates of MTB culture positive subjects and different prevalence rates of RIF resistance among MTB culture positive subjects are shown in Table 5. These calculations are based on hypothetical prevalences and the overall sensitivity and specificity of Xpert RIF resistance detection observed in all four clinical studies (sensitivity 94.7% and specificity of 99.0%). In the US population with TB the prevalence of rifampin resistance is approximately 1.8% [14].

**Table 5:** Hypothetical Predictive Values of Xpert detection of RIF resistance vs. DST

Prevalence of MTB culture positive	Prevalence of RIF resistance among MTB culture positive	Probability of RIF Resistance among Xpert results “MTB detected RIF Resistance detected”	Percent of Xpert results “MTB detected RIF Resistance detected” in the population	Probability of RIF Resistance among Xpert results “MTB detected, RIF Resistance not detected”
5%	1.0%	48.4%	0.09%	0.04%
	1.5%	58.6%	0.11%	0.06%
	2.0%	65.5%	0.13%	0.08%
	10%	91.2%	0.47%	0.45%
	50%	98.9%	2.17%	3.39%
*11.8%*	1.0%	48.4%	0.21%	0.05%
	*1.5%*	*58.6%*	*0.26%*	*0.07%*
	*2.0%*	*65.5%*	*0.31%*	*0.10%*
	10%	91.2%	1.11%	0.51%
	50%	98.9%	5.11%	4.16%
20%	1.0%	48.4%	0.35%	0.05%
	1.5%	58.6%	0.44%	0.07%
	2.0%	65.5%	0.52%	0.10%
	10%	91.2%	1.88%	0.54%
	50%	98.9%	8.66%	4.46%

## Discussion

We found high G4 Xpert sensitivity and specificity for detection of MTBc and RIF resistance in both analytical and clinical specimens collected from TB culture positive and negative subjects from US and non-US settings. Sensitivity for detection of MTBc in clinical samples was 99.7% among smear-positive specimens, 76.1% among smear-negative specimens and specificity was 98.7%. These results are very similar to the 2013 Cochrane review which found sensitivity of 98% and 67% for smear positive and smear negative specimens respectively, and a pooled specificity of 98% among studies using prior versions of the assay [4]. Xpert sensitivity and specificity for RIF resistance detection was 94.7% and 99% respectively in our combined clinical studies. This also compared well to reports using prior Xpert versions (94% and 98%, respectively [4]). In our analytical study, sensitivity and specificity for RIF resistance detection was 97.7% and 90.8%, respectively. However, this changed to 97.7% and 99.0%, respectively, when DNA sequencing was used as the reference method. Several recent studies have shown that some *rpoB* mutations are associated with low-level RIF resistance that are not detected by phenotypic testing, yet have poor clinical outcomes equivalent to patients with high level RIF resistance [10–13].

A small study by Sohn et al. [15] evaluated the diagnostic accuracy of both G4 and the previous version of the Xpert assay using induced sputum samples from 502 subjects in Montreal, Canada. The overall sensitivity of Xpert was very low: 11/25 (46%) overall, 6/7 (86%) for smear-positive specimens, and 5/17 (29%) for smear-negative specimens. Sensitivity of the G4 (5/15, 33%) was observed to be lower than the previous version (6/10, 60%), but this difference was not significant and was not stratified by smear status. The authors hypothesized that the lower sensitivity may have been related to lower bacillary load at presentation. Another possibility is that target bacilli may have been diluted by the saline used for induction [16].

We observed a statistically significant decrease in sensitivity among induced sputum samples compared to expectorated sputum for AFB smear-negative subjects (4/10 vs. 77/97; *p*-value <0.01), but not in smear positive samples (15/15 induced, 285/286 expectorated). However, other studies have observed that induced sputum samples had a higher diagnostic yield by culture [17] and shorter times to positivity by MGIT [18].

Thirteen (1.2%) of 1,126 specimens had a non-determinate Xpert result, which was lower than previously reported rates of >5% using earlier Xpert versions. Four samples were determined to be RIF susceptible by DST and RIF resistant by Xpert and three were determined to have RIF resistance-associated mutations in the *rpoB* core region by bi-directional sequencing although one was a non-resistance associated silent mutation. This leads to a 0.94% (4/427) false positive rate, or 0.47% (2/427) if using sequencing based mitigation.

There are several limitations to this study. First, sample collection, processing, shipment, storage and testing were done across various settings, which may have introduced some variability to our data collection and sample processing methods. In particular, one of the clinical studies was conducted as part of a research study, while the others analyzed leftover SOC specimens. For this reason the research clinical study was analyzed separately (see Additional file 1) and a poolability analysis was conducted to demonstrate that the four clinical studies could be combined. In addition, some specimens had been frozen and stored prior to testing, which may have introduced variability in testing results.

## Conclusions

We found that the G4 Xpert assay had low rates of non-determinate and false positive RIF resistance results that were not consistent with previously reported rates observed at some sites. In addition, we found high sensitivity and specificity for MTBc and RIF resistance detection that compared well to the previous versions of the assay [4]. Most published reports of Xpert assay performance were conducted using the previous versions of the assay; our findings represent one of the first large studies reporting G4 Xpert assay performance and add to the growing literature [19–22] in both high- and low-TB prevalence settings.

## Additional file

Additional file 1: Supplementary Materials. (PDF 239 kb)

## Abbreviations

CS1: Clinical study 1
CS2: Clinical study 2
CS3: Clinical study 3
CS4: Clinical study 4
NJMS: New Jersey Medical School
NPV: Negative predictive value
PCR: Polymerase chain reaction
PPV: Positive predictive value
RIF: Rifampin
SOC: Standard of care
TB: Tuberculosis
WHO: World Health Organization
Xpert: Xpert^®^ MTB/RIF
