# Supplementary material for: Performance of the G4 Xpert® MTB/RIF assay for the detection of Mycobacterium tuberculosis and rifampin resistance: a retrospective case-control study of analytical and clinical samples from high- and low-tuberculosis prevalence settings
Source: BMC Infect Dis. 2016 Dec 20;16:764. doi: 10.1186/s12879-016-2039-4 (PMC5168809; doi:10.1186/s12879-016-2039-4)
Supplement: Supplementary file 1 — Supplementary Materials. (PDF 239 kb) [file 12879_2016_2039_MOESM1_ESM.pdf]

Supplemental Materials

Methods

Study design and specimen inclusion criteria – Clinical studies

Clinical study 1 (CS1)

CS1 consisted of four collections. Collection one samples included 176 culture-positive and -negative unprocessed sputum samples from Lima, Peru. These samples were obtained from subjects enrolled at the Tropical Medicine Institute Alexander von Humboldt, Universidad Peruana Cayetano Heredia from November 2010 to January 2011. Collection two samples included 400 culture-positive unprocessed sputum samples from Cape Town, South Africa. Collection two samples were obtained from subjects enrolled by the Institute for Infectious Diseases and Molecular Medicine, University of Cape Town in South Africa from November 2010 to January 2011. These samples were excluded due to insufficient volume. Collection three samples included 400 partially processed (see below) and archived culture-positive specimens prospectively collected from HIV-negative adults in Vietnam, South Africa and Bangladesh. These were obtained from subjects enrolled by the Pham Ngoc Thach Hospital in Vietnam from April 2007 to April 2009, the South African Medical Research Council in South Africa from May 2009 to May 2010, and the International Centre for Diarrheal Disease Research in Bangladesh from May 2006 to May 2007. Collection four samples included 50 partially processed (see below) MTB culture-negative sputum specimens obtained from HIV-negative adults from South Africa and Vietnam. Collection 4 specimens were tested among Collection 3 specimens to provide a control group and minimize the risk of bias in testing known populations of positive specimens. Two sputum samples were collected from each study participant; the specimen provided for Xpert testing was based on sufficient volume available.

Symptoms suggesting pulmonary TB were defined as: persistent cough generally ≥2 weeks and at least one other symptom suggestive of TB such as fever, malaise, recent weight loss, night sweats, contact with an active TB case, hemoptysis, chest pain, loss of appetite.

Samples in Collection three and four were partially processed prior to Xpert testing by homogenizing for one minute with a vortexer and glass beads and split into 500 µL aliquots. Aliquots underwent microbiologic testing for MTB and were frozen at -70ºC until shipment on dry ice to NJMS for testing by Xpert. Although partially processed, these specimens were treated in the Xpert as if they were raw sputa.

Clinical Study 2 (CS2)

Up to 3 specimens were collected from each patient for MTB diagnosis. Subjects with at least one culture positive specimen were consecutively selected for Xpert testing using the first specimen with sufficient volume. MTB negative specimens were selected by choosing the first available study participant with all MTB negative baseline cultures that were collected after an eligible MTB positive case. An additional 25 MTB negative cases were randomly selected and included for testing throughout the study.

Clinical Studies 3(CS3) and 4 (CS4)

These studies are described in the main paper.

Sample inclusion criteria

Criteria for inclusion of samples in this analysis from CS1 included: 1) subject was not on TB treatment at the time of collection; 2) sufficient sample volume was available for Xpert

testing; and 3) results from AFB smear, culture, MTB identification and phenotypic DST results (if MTB culture positive) were available; for specimens with no DST results, an additional sputum sample was tested for DST if available. Criteria for inclusion of samples from CS2, CS3 and CS4 included: 1) subject was newly suspected to have pulmonary TB (at least 6 months from the last diagnosis of TB); 2) sufficient sample volume was available for Xpert testing; and 3) AFB smear, MTB culture and DST results (if MTB culture positive) were available.

Laboratory testing - Analytic study

The culture isolates were spiked at known concentrations of colony forming units (CFU)/mL into aliquots of a pool of sputa known to be negative for MTB and for nontuberculous Mycobacterium. Forty percent of the isolates were spiked at low concentrations near the assay cutoff (1500 CFU/mL), 30% at moderate concentrations (15,000 CFU/mL) and 30% at high concentrations (150,000 CFU/mL).

Statistical Analysis

Results for detection of MTBc and RIF resistance were defined as: 1) true positives (TP) if they were positive by Xpert and positive by culture/DST; 2) false positives (FP) if they were positive by Xpert and negative by culture/DST; 3) false negatives (FN) if they were negative by Xpert and positive by culture/DST; and 4) true negatives (TN) if they were negative by Xpert and negative by culture/DST. Sensitivity was defined as TP/(TP+FN) and specificity as TN/(TN+FP).

Sample size for MTB detection using the Xpert MTB/RIF Assay relative to Culture.

Sample Size Analysis for Sensitivity for MTB Detection

H_0: Sensitivity = 95% (relative to culture procedure in protocol)

H_A: Sensitivity < 90% (relative to culture procedure in protocol)

With power > 90% to reject the Null if Alternate is True

Minimal Sample Size = 400, Critical Value ≤ 27 FN (or lower 95% 2-sided CI ≥ 90 if SS > 400)

The actual probability calculations are as follows:

Equation-1 $$P(FN \leq 27 \mid Null) = \sum_{d=0}^{d=27} \binom{400}{d} 0.95^{400-d} 0.05^{d} = 95.2\%$$

Which is the probability of passing given the Null Hypothesis is true.

Equation-2

$$1 - P(FN \leq 27 \mid Alternate) = 1 - \sum_{d=0}^{d=27} \binom{400}{d} 0.90^{400-d} 0.10^{d} = 98.5\% = Power$$

Sample Size Analysis for Specificity for MTB Detection

H_0: Specificity = 98% (relative to culture procedure in protocol)

H_A: Specificity < 95% (relative to culture procedure in protocol)

With power > 95% to reject the Null if Alternate is True

Minimal Sample Size = 500, Critical Value ≤ 15 FP (or lower 95% 2-sided CI ≥ 95 if SS>500)

The actual probability calculations are as follows:

Equation-3 $$P(FN \leq 15 \mid Null) = \sum_{d=0}^{d=15} \binom{500}{d} 0.98^{400-d} 0.02^{d} = 95.3\%$$

Which is the probability of passing given the Null Hypothesis is true.

Equation-4

$$1 - P(FN \leq 15 \mid Alternate) = 1 - \sum_{d=0}^{d=15} \binom{500}{d} 0.95^{500-d} 0.05^{d} = 98.0\% = Power$$

Sample Size

The sample size required for the sensitivity and specificity analyses is 400 MTB culture positive and 500 MTB culture negative specimens, respectively. Our study included 468 MTB culture positive specimens and 628 MTB culture negative specimens. Thus, the sample size requirement was met for both sensitivity and specificity for the detection of MTB using the Xpert MTB/RIF Assay.

Sample size for detection of RIF resistance.

Achieving sample size for rifampin resistance was not expected because of the infrequency of rifampin resistance among clinical isolates. Therefore, we conducted the analytic study by testing spiked samples.

Results

CS1 results

Among the 531 samples included in the analysis, 366 were culture positive and 165 were culture negative. Overall, Xpert detected 345 of 366 culture positive specimens for a sensitivity of 94.3% (95% CI: 91.4-96.2%). Xpert detected MTB in 270 of 271 smear positive, culture positive cases for a sensitivity of 99.6% (95% CI: 97.9-99.9%) and 75 of 95 smear negative, culture positive cases for a sensitivity of 78.9% (95% CI: 69.7%-85.9%). Overall, Xpert did not detect MTB in 161 of 165 culture negative specimens, for a specificity of 97.6% (95% CI: 93.9%-99.1%).

Among the 17 samples with RIF resistance on DST, one sample was Xpert MTB negative. Xpert detected RIF resistance in 15 of 16 samples for a sensitivity of 93.8% (95% CI

71.7-98.9). Xpert did not detect RIF resistance in 314 of 322 specimens that were negative by DST for a specificity of 97.5% (95% CI 95.2-98.7). Bi-directional sequencing was performed on all culture positive isolates with discrepant results. There were 21 MTB culture positive specimens that were MTB not detected by Xpert; sequencing confirmed all to be MTB positive. There were nine samples with discordant RIF resistance results; sequencing confirmed results of the Xpert Assay in 3 of the 9 discordant specimens (Table 1).

Poolability Analysis

The four clinical studies were tested for homogeneity by analyzing the results across multiple parameters using the Fisher's Exact Test. A critical p-value was set to 0.01 due to multiple testing in several categories (Bonferroni principle). No differences were identified across the following parameters (p>0.01): protocol, archived or fresh samples, processed or unprocessed samples, gender, age group and site (data not shown). Some difference was found for sensitivity across site (p=0.08), specificity across site (p=0.06) and specificity across US vs. non-US samples (p=0.04) but these differences did not meet the cutoff for significance. Sensitivity was different across sample collection method (induced vs. expectorated, p=.0029). However, it was felt that the small number of induced samples relative to expectorated and the relative large percentage of false negatives in the induced samples yielded the small p-value. Forty percent (10/25) of the MTB positive culture induced specimens were AFB smear negative. Of these ten specimens, six were false negative by Xpert MTB/RIF Assay. The sensitivity may therefore have been lower in induced specimens due to the large percentage of AFB smear negative specimens in this population and the potential dilutional effect of the induction

process[1]. The studies were therefore determined to be poolable and the results of testing from all four clinical studies were combined and are presented together.

References

1. Theron G, Peter J, Calligaro G, Meldau R, Hanrahan C, Khalfey H, Matinyenya B, Muchinga T, Smith L, Pandie S *et al*: **Determinants of PCR performance (Xpert MTB/RIF), including bacterial load and inhibition, for TB diagnosis using specimens from different body compartments**. *Scientific reports* 2014, **4**:5658.

Table 1: Clinical Study 1 – discrepant Xpert assay and culture results confirmed by sequencing

**Culture/DST Result**	**Xpert MTB/RIF Assay Result**	**n**	**Bi-directional sequencing**
MTB positive/RIF-resistant	MTB NOT DETECTED	1	1 of 1 RIF resistant
MTB positive/RIF-susceptible	MTB NOT DETECTED	19	19 of 19 RIF susceptible
MTB positive/DST not done	MTB NOT DETECTED	1	1 of 1 RIF susceptible
MTB positive/RIF-resistant	MTB DETECTED; Rif Resistance NOT DETECTED	1	1 of 1 RIF resistant
MTB positive/RIF-susceptible	MTB DETECTED; Rif Resistance INDETERMINATE	4	4 of 4 RIF susceptible
MTB positive/RIF-susceptible	MTB DETECTED; Rif Resistance DETECTED	4	3 of 4 RIF resistant<br>1 of 4 RIF susceptible
